# Innovative Solutions in the Design of Microfinishing Attachments for Surface Finishing with Abrasive Films

**DOI:** 10.3390/mi16020165

**Published:** 2025-01-30

**Authors:** Wojciech Kacalak, Katarzyna Tandecka, Zbigniew Budniak, Thomas G. Mathia

**Affiliations:** 1 Faculty of Mechanical Engineering and Energy, Koszalin University of Technology, 75-620 Koszalin, Poland; wojciech.kacalak@tu.koszalin.pl (W.K.); zbigniew.budniak@tu.koszalin.pl (Z.B.); 2 Laboratoire de Tribologie et Dynamique des Systemes (LTDS), Ecole Centrale de Lyon, Centre National de la Recherche Scientifique, 69134 Lyon, France

**Keywords:** microfinishing attachment, microfinishing film, metal finishing, surface finishing, abrasive film

## Abstract

The study introduces new technologies of microfinishing, which are primarily aimed at cylindrical surfaces but with machining effectiveness, precision, and surface longevity. In the newly proposed dual-zone microfinishing method, symmetrical abrasive film feeding systems are adapted with a lever mechanism and a pivoting pressing assembly to simultaneously conduct processing in two zones. With such a design, uniform force distribution is ensured, while mechanical deformation is reduced to raise the utility of the abrasive film and lower scraps for better economic performance. Also, the application of microfinishing operations combined with carbon layer deposition using graphite-impregnated abrasive films is introduced as a novel method. This process combines surface refinement and the forming of wear-resistant carbon coatings into one single operation, resulting in increased wear resistance and reduced forces of friction. Further stabilization of the conditions for microfinishing is achieved by immersing the processing zone in a fluid medium due to increased lubrication, improvement in heat dissipation, and the optimization of surface properties. It is particularly suitable for high-precision applications and a maintenance-free environment such as military, vacuum, and low-temperature systems. The experimental results show the effectiveness of the proposed methodologies, underscoring their ability to create remarkably smooth surfaces and very robust carbon textures simultaneously.

## 1. Introduction

**Figure 1:**
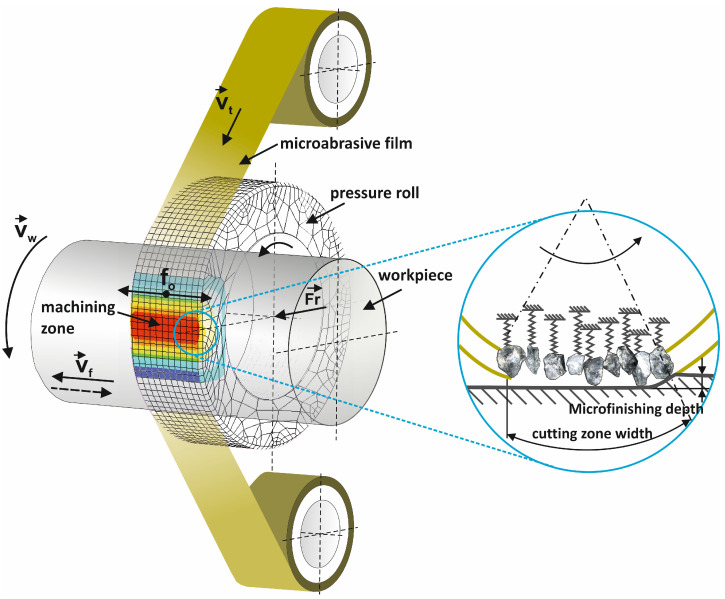
Kinematic diagram of rotary surface finishing using microabrasive films, where the following quantities are indicated on the diagram: vt—tool speed, vw—workpiece speed, vf—tool feed speed, fo—tool oscillation frequency, and Fr—the pressure force of the pressing roller [1].

Microfinishing with abrasive films is predominantly performed using attachments mounted on conventional machine tools, such as lathes. The attachment is typically mounted on the cross slide. It includes a feed roller to supply the film, a pressure roller to apply the film to the workpiece, and a driven roller to pull and collect the film (Figure 1). This arrangement ensures the abrasive film’s transport to the microfinishing zone, its application against the workpiece, and its removal along with processing residues. Abrasive films are primarily used for external cylindrical and flat surfaces and less frequently for internal bores. Key processing parameters affecting the surface quality include the circumferential speed of the workpiece, feed rate, film traverse speed, film pressure against the workpiece, and the hardness of the applied pressure roller. Additionally, to increase the density of finishing traces, an oscillatory motion of the abrasive film with an adjustable frequency is used, which adds complexity to the attachment design. Processing can be performed in a concurrent manner, where the velocity vectors of the workpiece and film movement are aligned, but counter-directional processing is more commonly employed, as it delivers superior results.

Pressure application is very critical for the accuracy and effectiveness of the microfinishing operation, especially in the case of cylindrical surfaces [2,3]. The quality of the surface finishes, material removal rates, and efficiency of the whole operation are closely linked to the pressure dynamics occurring during the microfinishing operation [4,5,6]. The complete understanding and optimization of the dynamics enable consistent high-quality results on a variety of materials with complex geometries [7,8,9]. The elastic pressing mechanisms are, therefore, designed in such a way that a uniform distribution of pressure between the abrasive film and the workpiece is ensured [10,11,12]. Due to the inherent elasticity in the roller, small-scale surface discrepancies are seamlessly adjusted; thus, the contact remains constant, and localized stress concentrations are avoided, a significant issue in microfinishing applications where precision and consistency have extremely high demands [13,14,15]. The hardness of the pressing roller, usually measured in terms of the Shore A scale, must be chosen carefully depending on the features of the workpiece material and the desired surface finish [16,17,18]. This deliberation creates a balance between effective material removal and preserving the durability of the abrasive film [19,20,21]. The microfinishing zone, where the abrasive film contacts the workpiece, is characterized by several critical factors [22,23]. A uniform pressure distribution within this area is of the utmost importance for achieving constant material removal and a homogeneous surface texture [24,25]. Irregularities in the pressure could result in surface defects such as waviness or localized overheating, thereby affecting the quality of the finished surface [26,27,28,29]. Roller shape, the elasticity of the materials, and the force exerted have a very important configuration in the contact region, which may lead to the optimization of effectiveness in the abrasive process [30,31,32].

The temperature in the microfinishing zone is a very important factor of the process. Because of the frictional interaction between the workpiece and the abrasive film, heat is evolved, which harms both the abrasive film and the workpiece surface being processed [28,33,34]. Extremely high temperatures may result in thermal damage to the workpiece material, such as hardening or softening, or decrease the cutting efficiency of the abrasive grains. In order to help minimize this effect, elastic rollers ensure an even pressure distribution and also help to reduce localized heating [34,35,36,37]. Additionally, fluid immersion cooling, in some cases air jets, provides better temperature control. Fluid immersion, in particular, offers many benefits in terms of heat dissipation, lubrication, and removing particles from the contact zone, resulting in a cleaner, more efficient microfinishing process [19,38,39]. When pressure is optimized in elastic pressing mechanisms, the advantages trickle down to every nook and cranny of the process. Superior surface quality is achieved through better and smoother surfaces that are characterized by low roughness and uniform texture [22,40,41]. This results in an improvement in the process efficiency, as material removal rates are optimized, and the cycle times are reduced [8,42]. It also extends the life of the abrasive film and pressing roller since localized stress and heat generation are reduced [43,44]. The result of this, hence, is lower operating costs and an increase in process sustainability. The ability to adapt to a wide range of materials and geometrical configurations is one of the most striking features of elastic pressing mechanisms, which makes them so pertinent to contemporary manufacturing [45]. To address challenges associated with consistent pressure distribution, thermal effects, and compatibility with materials, this system realizes precise and high-quality surface finishing in many industrial applications [7,46,47,48]. The demand for precision and durability in surface treatment applications underscores the vital role of elastic pressing mechanisms within microfinishing techniques. At the same time, these mechanisms can be made to break the norms of surface finishing and set new standards for efficiency and quality of manufacturing processes through design improvement and real-time monitoring.

**Figure 2:**
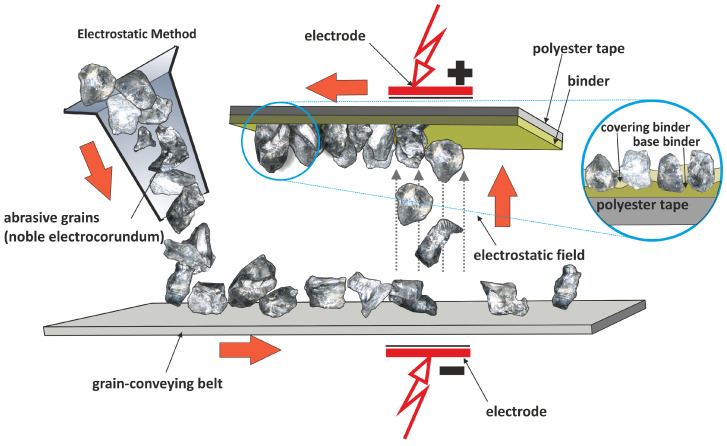
The production scheme in the electrostatic field of a microfinishing film [49].

Abrasive films used for microfinishing are classified into two groups. The first group comprises films for preliminary finishing, characterized by higher efficiency in reducing surface roughness due to the electrostatically oriented grains on the binder surface, which maximize the number of active cutting edges (Figure 2).

**Figure 3:**
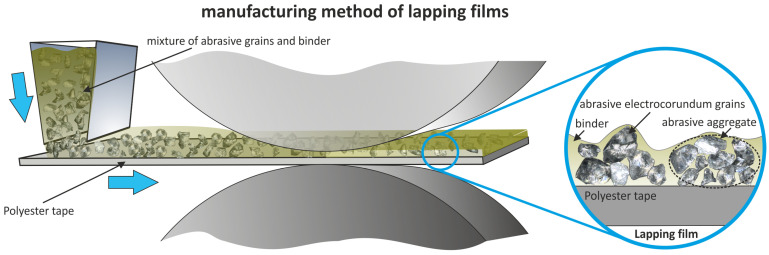
Schematic depicting the production of lapping film [1].

The second group includes abrasive films for microfinishing, in which abrasive grains are applied to a polyester film substrate using a gravitational method and further coated with a thin binder layer (Figure 3). In lapping processes, films with grains made from fused alumina or synthetic diamond are typically used. Very smooth surfaces can be achieved through sequential processing with abrasive films of decreasing grain sizes. Preliminary finishing uses films with electrostatically applied grains of a nominal size of 30 µm, which are replaced step-by-step with films containing smaller grains that are used only once. The authors of the study investigated the byproducts of the microfinishing process and observed that a significant portion of the abrasive material becomes dislodged from the processing zone, potentially causing deep scratches on the finished surface [1,50]. To address this issue, the authors proposed abrasive tools with discontinuous surfaces, which effectively eliminate this phenomenon [49].

Depending on the technological requirements of the surface, to achieve the highest precision, the process concludes with the use of microfinishing films featuring a specific grain size, followed by the film being guided over a directing roller into the processing zone. Then, it is pressed against the workpiece by a pressure roller, typically actuated by a pneumatic cylinder, and subjected to oscillatory motion using a mechanism that enables adjustable oscillation frequency. After processing, the film exits the finishing zone through a guiding roller and is wound onto a driven collecting roller, which also serves as a storage unit for the used abrasive film. In this setup, the workpiece is mounted on the lathe spindle, while the attachment is fixed to the lathe across the slide or table.

The solution developed by Supfina Grieshaber GmbH & Co. KG, Wolfach, Germany, introduces a system where the processed shaft is not only rotated but also oscillated. The abrasive film is fed into the processing zone and pressed against the rotating shaft on both sides by shaped sliding pressure pads. Above the processed workpiece, a rotating roller acts as a tensioning element for the film. Simultaneous processing in two zones increases processing efficiency.

Another innovation by Supfina in abrasive film processing is the method of centerless finishing for external cylindrical surfaces. Two guiding rollers placed side by side, driven in rotation, provide the workpiece with rotational motion. The abrasive film, which simultaneously performs oscillatory motion, is pressed against the workpiece by a third roller, ensuring the necessary constraints on the workpiece during the process.

Another category of equipment used for abrasive film microfinishing includes stationary machines. These are specialized or universal finishing machines with steplessly adjustable speeds for all motions, oscillation frequencies, processing pressures, and programmable cycle times. Specialized machines include those with microfinishing crankshafts that use abrasive films. In this case, no feed motion is utilized; instead, plunge finishing is performed. The workpiece is encircled by an abrasive film, which is pressed against the entire circumference of the workpiece as it rotates.

The need for high-precision surface finishing led to the development of high-precision microfinishing processes. Improvements in efficiency and surface quality, along with the customization of technologies to meet the diversifying needs of industry today, remain a challenge. In this paper, the challenges are addressed by showing new ideas in microfinishing technologies patented by the authors themselves. The main purpose of this study is to explain novel technological approaches, including dual-zone processing systems by combining microfinishing processes with carbon layer deposition, and complex configurations using abrasive films in a fluid medium. This paper points out the benefits brought about by these methods, especially in the area of balanced distribution of forces, extension of the life of surfaces, and the optimization of process parameters. Novel methodologies developed in this work open up new possibilities for surface treatment technologies, improving quality and performance in many industrial applications.

Despite significant advances in microfinishing technologies, existing solutions often fail to make a perfect compromise between efficiency, accuracy, and cost-effectiveness. Traditional single-zone microfinishing methods are typified by the limited force distribution and material removal uniformity, which can lead to mechanical distortion and lower surface quality. Additionally, the traditional abrasive film processing method is typified by the lack of an integrated approach that optimizes tool life and maintains a uniform finishing output. The new methods covered in this study address the problem areas with two-zone microfinishing using a pressure control lever system, fluid medium microfinishing, and carbon layer deposition during finishing. These new methods improve force distribution and film usage as well as extend the microfinishing applicability to a wider variety of materials and industrial processes. This research introduces a new paradigm in surface finishing technology by bringing together conventional processes and modern accuracy demands, thereby enabling enhanced performance, reducing material loss, and offering better surface characteristics.

## 2. Materials and Methods

The microfinishing process consisted of three consecutive steps, with each step employing an abrasion film with continuously reducing nominal grain sizes of 30, 15, and 9 μm. The duration of each step was 60 s, and the whole process took 180 s. The surface condition was from the turning operation. The test was performed on the GW1 microfinishing attachment (Koszalin University of Technology, Koszalin, Poland) on a superalloy Nimonic 80A shaft. Pressure was applied using a roller presser with a hardness of 50 Shore degrees and an applied force of 50 N. The abrasive film was supplied at 160 mm/min, while the workpiece was rotated at a surface speed of 40 m/min. In addition, the tool was also subjected to an oscillatory motion of 80 Hz in a bid to enhance the finishing action.

During the design process of microfinishing attachments, the software SolidWorks 2019 was utilized. The process of modeling innovative designs for devices used in smoothing with abrasive films employed CAD/CAE computer techniques using the SolidWorks software (SolidWorks version 2019). This software offers unlimited design possibilities by integrating advanced tools, such as part modeling, assemblies, and drawing functions, with built-in solutions for simulation, photorealistic visualization, animation, and motion simulation. The creation of 3D models primarily involved solid modeling operations, focusing on the modeling of individual parts and advanced assemblies. The design work was carried out in stages, starting with the initial modeling of individual parts and their assembly and concluding with motion analysis using the Motion Study add-in. The software also enabled an impressive photorealistic visualization of the project through the additional PhotoView360 module, operating within the SolidWorks environment.

## 3. Results and Discussion

### 3.1. Conventional Solution of the Microfinishing Attachment

**Figure 4:**
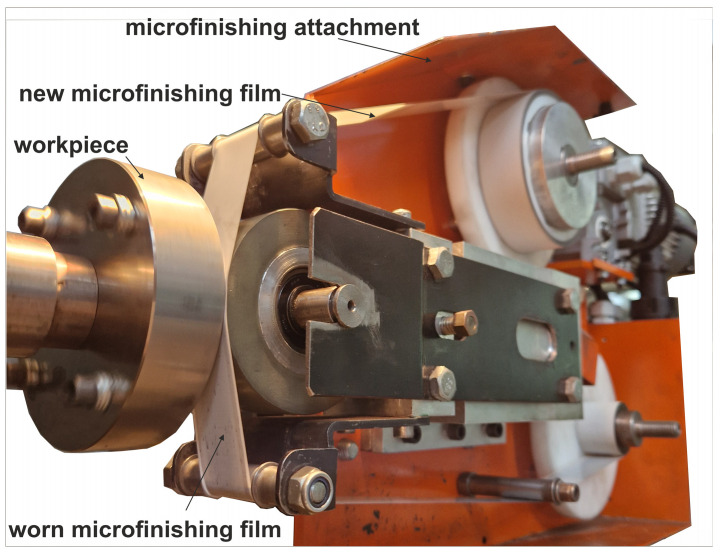
Research setup for the superfinishing process using abrasive films [51].

The GW-1 attachment was developed at the Koszalin University of Technology (Figure 4). This attachment is mounted in the tool holder of a lathe. It features separate drives for the abrasive film’s feed and oscillatory motion. The rotational speed of the workpiece is provided by the lathe spindle, while the attachment’s movement is facilitated by the lathe’s cross slide.

**Figure 5:**
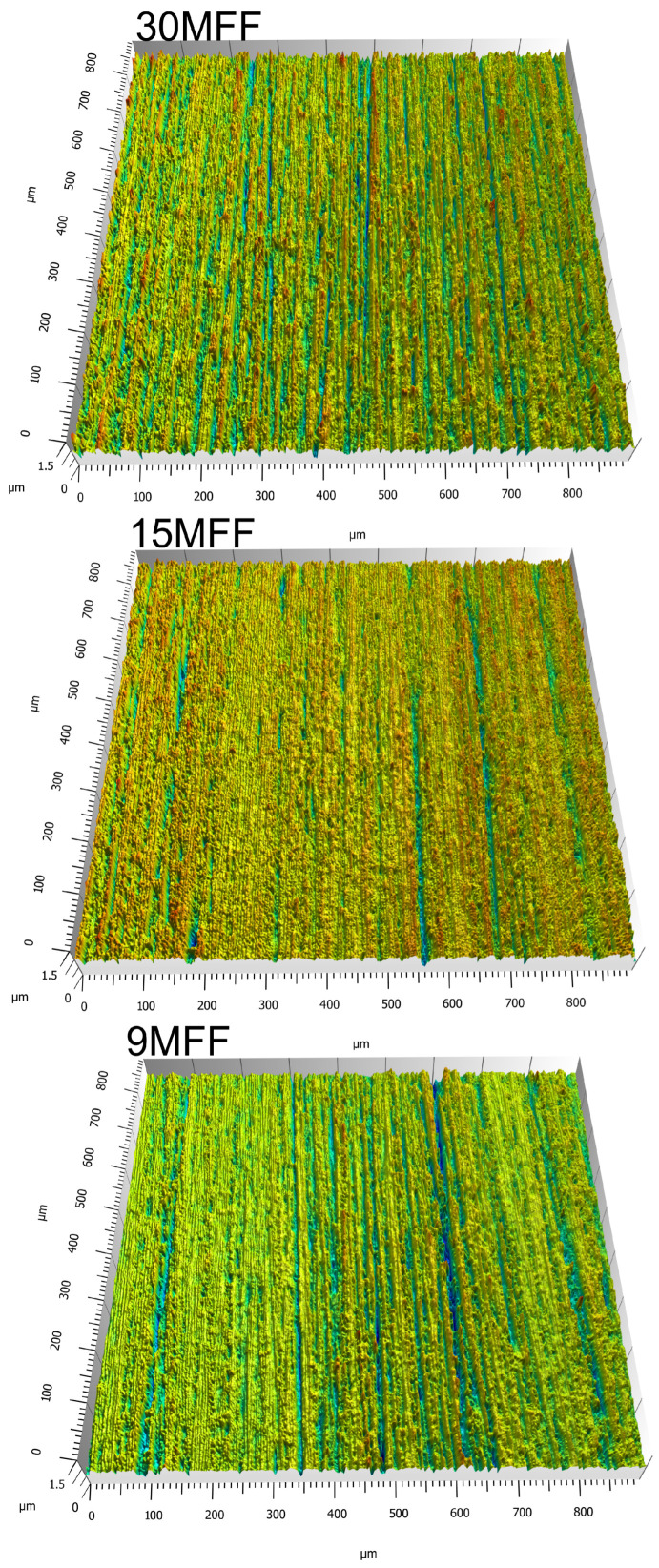
Images of surface topography in a 3D layout of the workpiece (Nimonic 80A) after the microfinishing process with abrasive films [52].

Abrasive film microfinishing is a highly efficient method for treating difficult materials such as the Nimonic 80A alloy [52]. The nickel superalloy is finding widespread applications in aerospace and power sectors due to its ability to withstand high temperatures, creep deformation, and oxidation. Machining processes used on the material are difficult due to its hardness and wear resistance, which makes processes such as grinding less efficient due to high cutting forces and undesirable thermal effects. The effectiveness of microfinishing with abrasive films is demonstrated in this research through a Nimonic 80A alloy shaft. Figure 5 shows the three-dimensional surface topography after microfinishing with abrasive films of different nominal grain sizes. This study demonstrates that the use of progressively finer abrasive films enables systematic improvement in reducing surface roughness and improving the overall surface quality.

**Figure 6:**
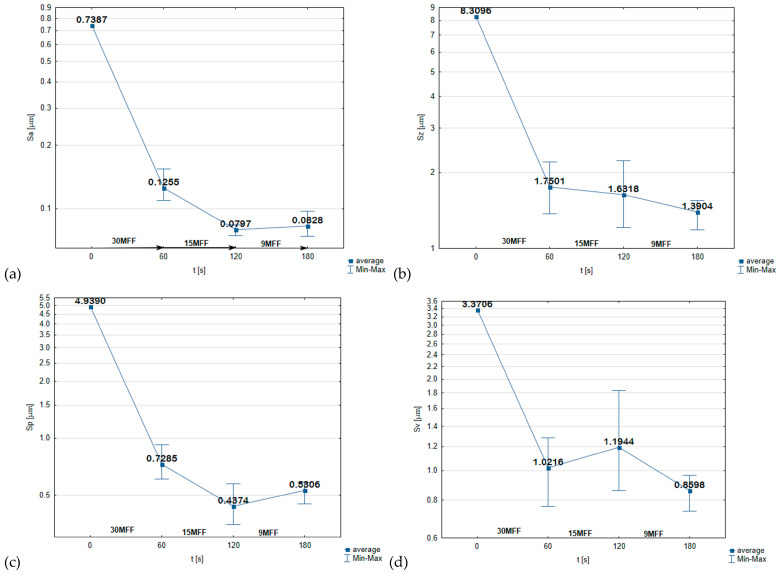
Surface roughness parameters for evaluating the surface roughness after the microfinishing process of Nimonic 80A: Sa—arithmetical mean height of the surface (**a**), Sz—maximum height of the surface (**b**), Sp—maximum height of peaks (**c**), and Sv—maximum height of valleys (**d**) [52].

The finishing quality can be quantified with surface roughness parameters given in Figure 6. The turned surface was of high irregularities prior to microfinishing, with a maximum height (Sz) of 8.3 µm. The application of an abrasive film with a nominal grain size of 30 µm removed about 6.5 µm of these irregularities, thus proving itself effective for removing gross imperfections. Further microfinishing operations involving 15 µm and 9 µm grain size abrasive films continued to reduce the roughness and yielded a highly smooth surface. Importantly, the 15MFF film removed peak irregularities, enhancing surface bearing capacity, with potentially very important tribological consequences for the machined material. The results achieved verify that the microfinishing process with the use of abrasive films is a viable process for machining hard-to-machine materials such that better surface quality can be achieved without causing severe thermal exposure or material structure degradation. In a subsequent study, the authors demonstrated that modifying the microfinishing head by offsetting the microfinishing zone from the workpiece axis increases the efficiency of the process. It is essential to explore such solutions that enhance the effectiveness of microfinishing operations [53]. To determine the efficiency of microfinishing, the authors developed a methodology based on the analysis of islands, which proves effective in evaluating surfaces after the abrasive process [50,54].

### 3.2. Assembly of Attachments for Two-Zone Abrasive Microfinishing with a Lever-Based Pressure Mechanism

**Figure 7:**
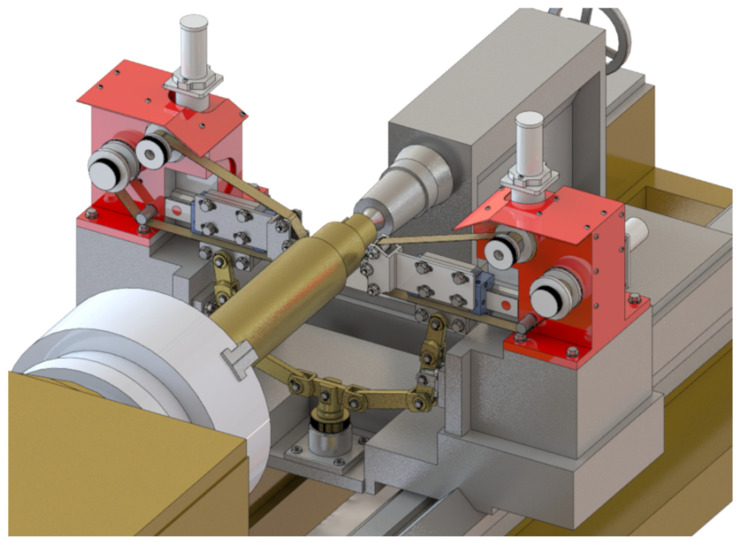
A microfinishing attachment for abrasive film finishing of external surfaces of workpieces [55].

The proposed technique presents a novel method for microfinishing external cylindrical surfaces using abrasive films in tape form. The machine setup consists of a cross slide having two independent mechanisms to feed the abrasive films that are placed symmetrically on either side of the workpiece. The lever mechanism-based device with pivoting assembly provides proper guidance and the efficient transmission of pressure from the abrasive film to the surface. It also features an integrated pressing mechanism for smooth operation with high accuracy. The described solution has been patented (Figure 7).

There is a common setup within the mechanical engineering of these mechanisms, whereby two, and in some cases three, rollers are aligned across a horizontal axis and mounted onto a rigid supporting structure with a view toward the conveyor abrasive films. In this arrangement, one roller is a source of supply for the unused abrasive film, and the other roller is the driven element for removing the film from the operation. In contrast, the device of the present invention enables the abrasive film to ride over rollers that are, at the same time, directed by the guiding mechanism and pressed upon by the pressing mechanism, forming a pivoting system. More precisely, the pressing roller engaging said pivoting mechanism is supported by a pair of guiding rollers carried on a double-arm lever.

**Figure 8:**
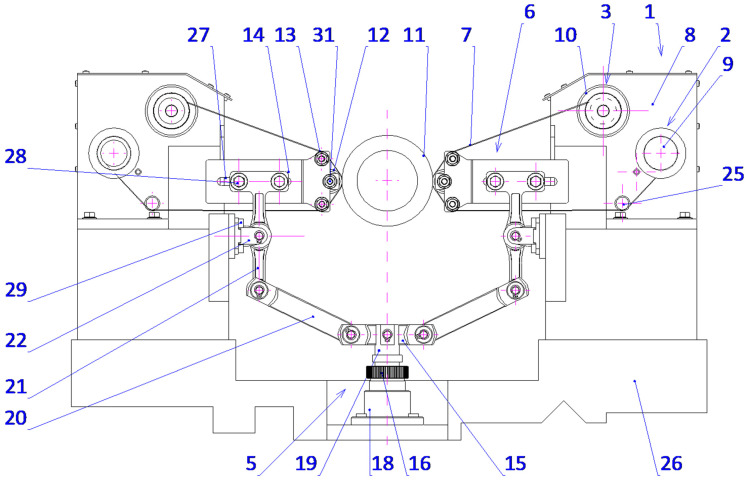
Side view of the head for microfinishing external cylindrical surfaces using abrasive films, featuring a lever mechanism and a pressing assembly.

**Figure 9:**
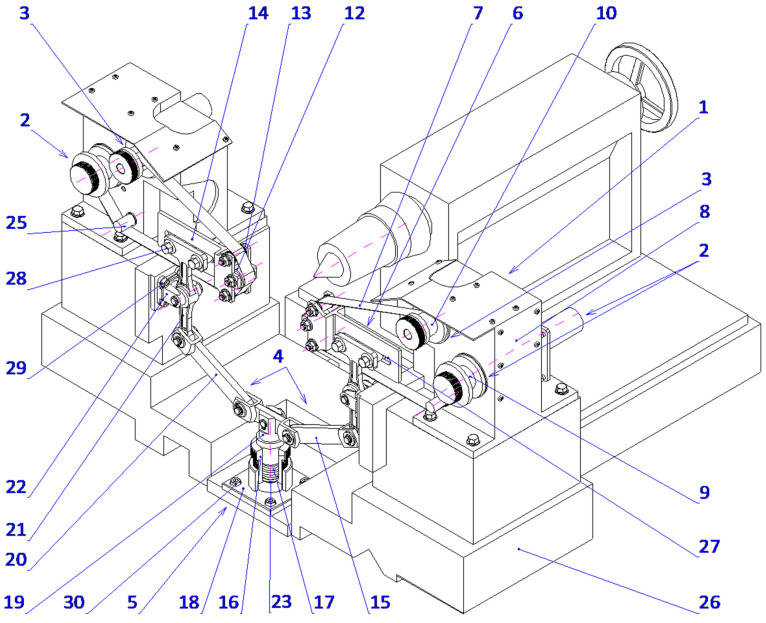
Isometric view of the head for microfinishing external cylindrical surfaces using abrasive films, showing the arrangement of the film feed systems, lever mechanism, and pressing assembly.

**Figure 10:**
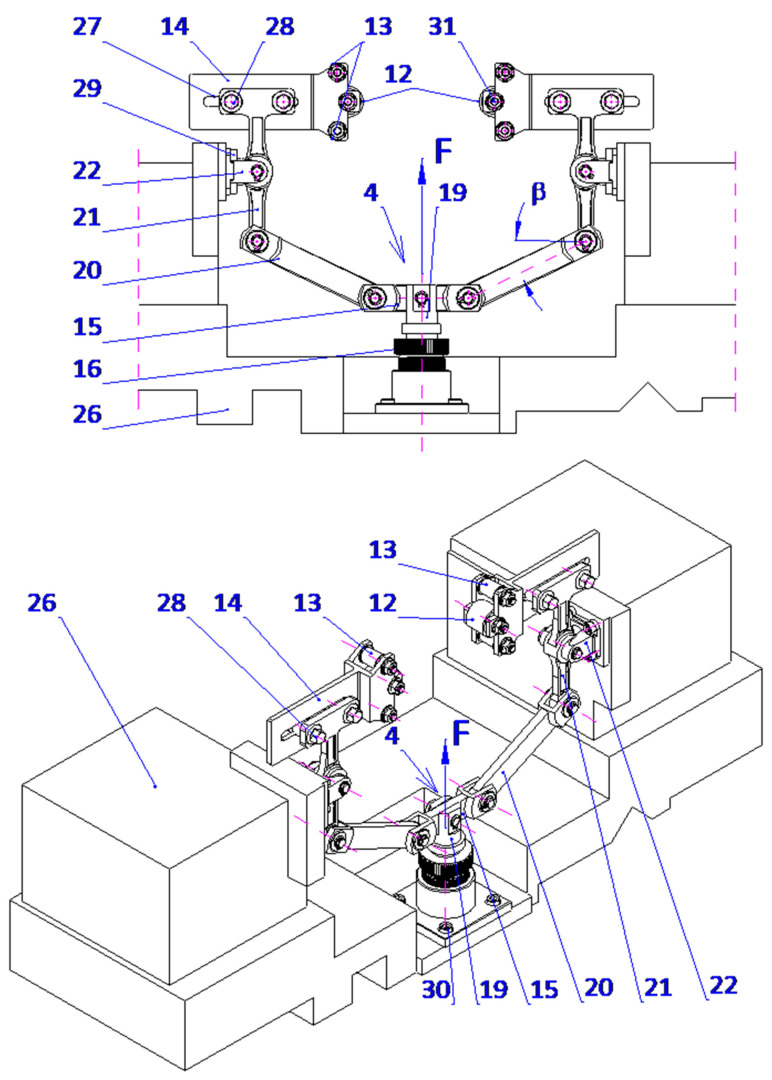
Lever mechanism and pressing assembly of the microfinishing head. View from the workpiece side, showing structural details and the arrangement of pressing elements.

**Figure 11:**
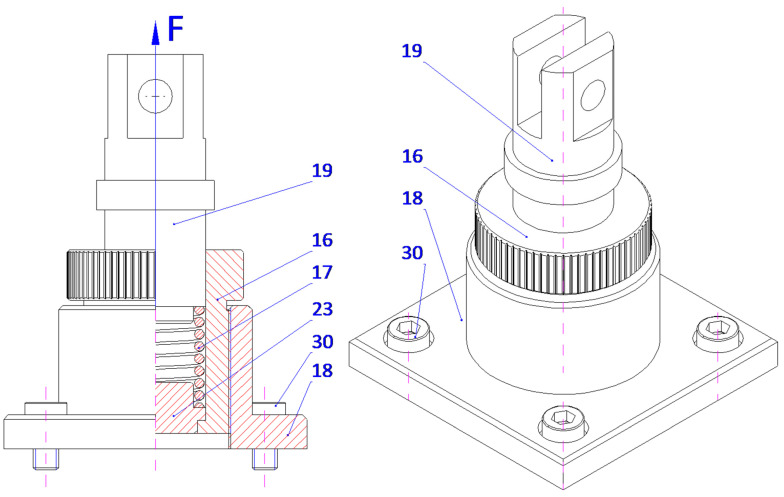
Axial cross-section of the pressing mechanism of the microfinishing head, showing structural details and the method for adjusting the pressing force of the abrasive film.

The lever is supported rotatably in the supporting structure around a predefined axis, whereby control over the applied force during pressing is possible as well as precise positioning of the pressing roller with respect to the work piece. This will ensure a stable interaction between the abrasive film and the workpiece surface, which is the most critical requirement for attaining maximum microfinishing efficiency. This leads to increased force exerted, dramatically outperforming others in conventional spring-loaded devices, which allows the user to perform microfinishing with much higher precision and control over the process. It is symmetrical on both sides of the workpiece and has an abrasive film supply system. Hence, the design allows for the possibility of simultaneous processing in two opposite areas. The setup increases the general efficiency of the process while assuring symmetrical distribution of forces and minimizing the probability of mechanical deformation in the machining setup. The mechanism of feeding is placed symmetrically and positioned in such a way that the abrasive film functions optimally, thereby reducing waste to a great extent and making the process economically more viable. This configuration, known for its striking flexibility, uses progressively decreasing grain size abrasive films as it moves to the final stages of surface finishing. The process, therefore, is excellent in creating very smooth surfaces, so it is specially adapted to the precision finishing of cylindrical, conical, and flat-ended workpieces. The process can be carried out on conventional lathes or on machinery designed specifically for oscillatory finishing. Compared with current technologies, the new process retains most of the advantages. Two-zone processing is, therefore, obviously capable of giving an opportunity to increase the efficiency drastically with a uniform surface quality over the whole workpiece. On the other hand, it dispels the necessity for independent power and control units, enabling a more compact design with reduced complexity, spatial needs, and often reduced integration efforts with parallel operating systems. The presented single-zone solution is a revolution for the industry of microfinishing, overcoming most disadvantages of traditional single-zone systems. However, it also substantially improves the effectiveness in terms of force application and film usage. It was envisioned that this solution would have graphical visualization, like Figure 8, Figure 9, Figure 10 and Figure 11, to take a much more holistic view of the design and its operational functionalities graphically. These graphical representations show the side, isometric, and cross-sectional views of the mechanism with novel attributes and operational efficiencies. In combination, some of the features of this unique machine have been recently added to enhance its ability to deal with problems concerning the exact microfinishing of cylindrical surfaces and hence enhance the efficiency and precision of the process. The designations used in this study are as follows: the abrasive film feed system (1), tape drive assembly (2), tape braking assembly (3), lever mechanism (4), pressing mechanism (5), abrasive film pressing assembly (6), abrasive film tape (7), housing of the abrasive film system (8), abrasive film winding roller (9), brake roller (10), workpiece (shaft) (11), pressing roller (12), guiding roller (13), guiding plate (14), fixed connector (15), adjustment sleeve (16), compression spring (17), support (18), movable pin (19), connector (20), double-arm lever (21), bracket (22), abutment (23), fork (24), guiding roller (25), cross slide (26), longitudinal slot (27), and screws (28, 29, 30).

The microfinishing attachment is equipped with two abrasive film feed systems (1), mounted on the cross slide (26) and symmetrically positioned relative to the workpiece (11), a lever mechanism (4), and a coupled pressing mechanism (5). Each abrasive film feed system (1) comprises two or three rollers connected to the housing (8), which is securely fixed to the cross slide (26). One of these rollers (10), part of the tape braking assembly (3), serves as the storage for the abrasive film, while the other, the driving roller (9), located in the drive assembly (2), collects the used abrasive film (7). The abrasive film passes between these rollers through the guiding roller (25) and the pivoting pressing assembly (6), which is attached to the double-arm lever (21) with screws (28). The movement of the pressing assemblies (6), as shown in Figure 3 and Figure 4, located on both sides of the processed shaft (11), is achieved by the rotation of the double-arm lever (21) around the axis of the support (22), which is secured to the cross slide (26) with screws (29). This assembly includes a plate (14) with a longitudinal slot (27) for screws (28) that determine its position. Additionally, the plate (14) features forks (24) that house the pressing roller (12), made of an elastomer on a metal axis, and two guiding rollers (13). The lever mechanism (4) includes a movable pin (19) cylindrically seated in the adjustment sleeve (16). At its upper end, the pin is firmly attached to a connector (15), which is pivotally connected on both sides to a link (20) that is inclined at a small angle (β), decreasing as the pin (19) extends.

Furthermore, the mechanism features a double-arm lever (21) pivotally mounted in the support (22). At one end, the lever is pivotally connected to the link (20), while the other end is attached to the abrasive film pressing assembly (6). The pressing force (F) is generated by the pressing mechanism (5), which includes a cylindrical support (18) with a flange at the bottom, securely fixed to the cross slide (26) with screws (30). A threaded hole in the support is connected to the adjustment sleeve (16), where a thrust spring (17) is housed. One end of the spring rests against the abutment (23) on the inner surface of the sleeve, while the other end applies force to the front surface of the pin (19), which fits snugly in the inner surface of the adjustment sleeve (16). The spring force (17) is pre-adjusted using the adjustment nut (16), continuously pushing the pin (19) axially forward.

The proposed method provides accurate, effective microfinishing of external cylindrical surfaces with abrasive films. Its novel design includes a counter-balanced mechanism for abrasive film feed combined with a lever-operated handle and a pressing assembly that performs processing simultaneously along two opposite zones. This, in turn, enhances the efficiency of the whole process, since it offers a fair distribution of forces and thus decreases the possibility of mechanical deformation. The grains of an abrasive film in this option are used optimally, which contributes to further waste reduction and economic efficiency. A lever system with pivoting pressing assembly ensures precise control over the applied force and ensures even contact between the abrasive film and the workpiece surface. The quality and precision are substantially better than with traditional spring-loaded systems. Due to this flexibility, the system is particularly well suited for the production of very smooth surfaces via the step-by-step application of abrasive films with a grain size that gets continually smaller. Its compact design does not require separate power and control units, reducing complexity, spatial requirements, and efforts for integration. That has overcome the key barriers to microfinishing by combining advanced mechanical design with improved operational efficiency. This method is especially suitable for implementation on conventional lathes and dedicated oscillatory finishing machines; hence, it imparts flexibility and adaptability for precision finishing operations across a spectrum of applications.

The proposed two-zone microfinishing process has several benefits compared to conventional single-zone processes. Among these advantages is the uniformity of forces imparted on the two sides of the workpiece, significantly reducing the occurrence of mechanical deformation. Force balance ensures uniform surface pressure, thereby increasing material removal uniformity and reducing stress concentrations that lead to defects. The second major advantage of this method is the economical utilization of the abrasive film. In standard single-zone microfinishing, much of the abrasive film can be inefficiently utilized, resulting in a higher wastage of materials. The two-zone configuration places wear uniformly over the two processing zones, thereby optimizing the abrasion life of the abrasive film and ensuring greater economic efficiency.

Additionally, the lever-driven pressing mechanism also enhances the microfinishing stability and accuracy. In comparison with conventional spring-type mechanisms that have pressure variations introduced by elastic deflection, the lever mechanism affords more controllable and repeatable force delivery. As such, this renders better surface evenness and minimal roughness value fluctuations. The arrangement mentioned enables improved efficiency in processing. With the combination of microfinishing used in two zones at the same time, the overall processing time is reduced, thereby making the system very useful for high-precision manufacturing processes where productivity becomes an important consideration. Additionally, the ability to precisely control the applied pressure enables the effective processing of materials with different hardness levels, thereby extending the application of the process to a wide range of engineering components. The newly developed two-zone microfinishing machine with a lever-operated pressure system is a significant advancement in the precision finishing process. It provides maximum force distribution, better utilization of the film, enhanced process efficiency, and better control of the surface finish. These merits of the developed system make it strongly suitable for industrial applications where a high-quality surface finish and high-dimensional accuracy are needed.

### 3.3. Microfinishing Attachment for Cylindrical Surfaces with a Processing Zone Submerged in a Processing Fluid

**Figure 12:**
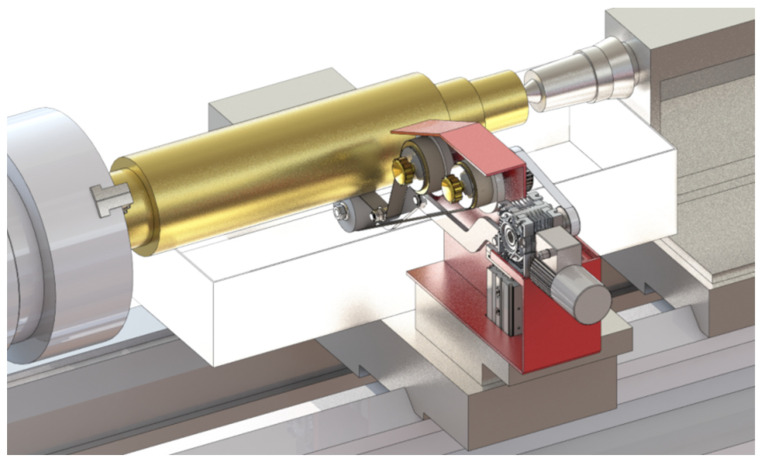
A device for abrasive film finishing of external cylindrical surfaces, with the processing zone immersed in a machining fluid [56,57].

The proposed solution includes immersing the processing zone in a processing fluid atmosphere. This is believed to increase the effectiveness of abrasive films, increase the machining efficiency, and improve the general process effectiveness. The processing zone, when placed under the processing fluid, results in a significant reduction in the influence of the ambient atmosphere on the physical phenomenon taking place inside the micro-cutting zone. It also provides the separation of effects due to the elements of the processing fluid on workpiece surface development, assuring a controlled and homogeneous finishing process. The outlined solution is protected by a patent (Figure 12).

The proposed process relates to the technical arrangement that is designed for the microfinishing of cylindrical outer surfaces using abrasive films within a processing space impregnated with a processing fluid. The device has a tank rigidly fixed to the base of the machine and the means for supplying abrasive films. Furthermore, two parallel rollers are mounted next to the housing of the feed mechanism. The first component is mounted on the cross slide of the apparatus. One of the rollers is a feeding mechanism for the abrasive film, while another is a drive roller that allows for the unwinding of the film in operating processes.

**Figure 13:**
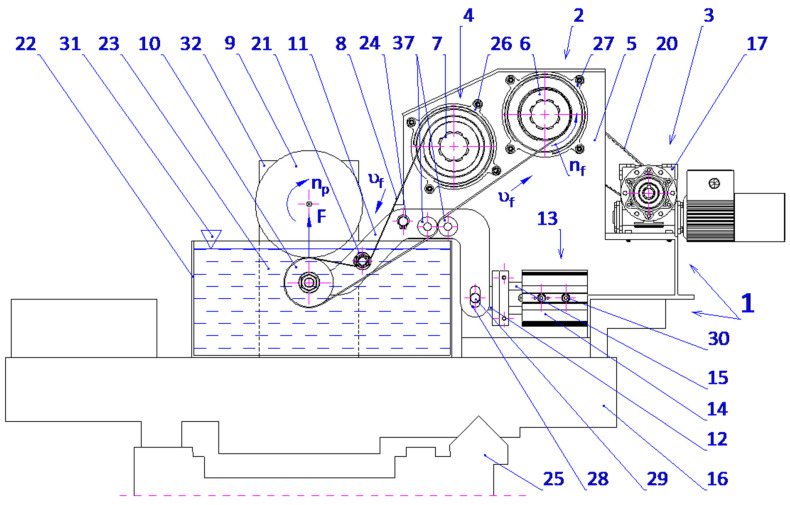
The attachment in a side view with the microfinishing zone submerged in the processing fluid.

**Figure 14:**
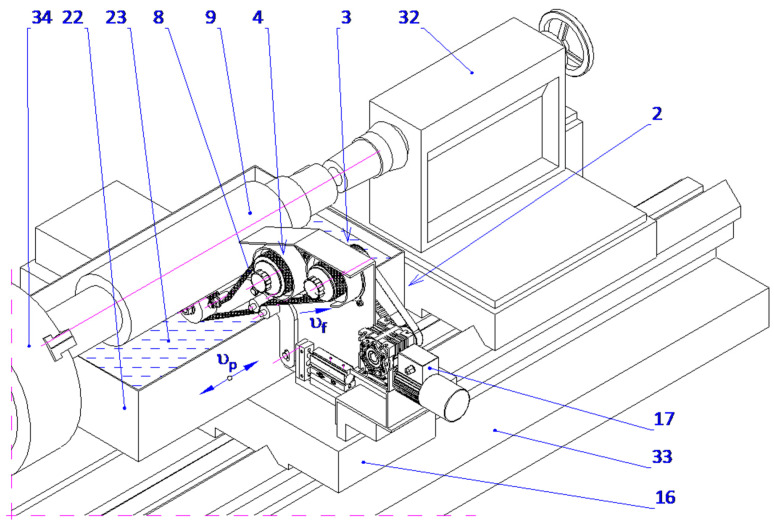
The microfinishing attachment in an isometric view.

**Figure 15:**
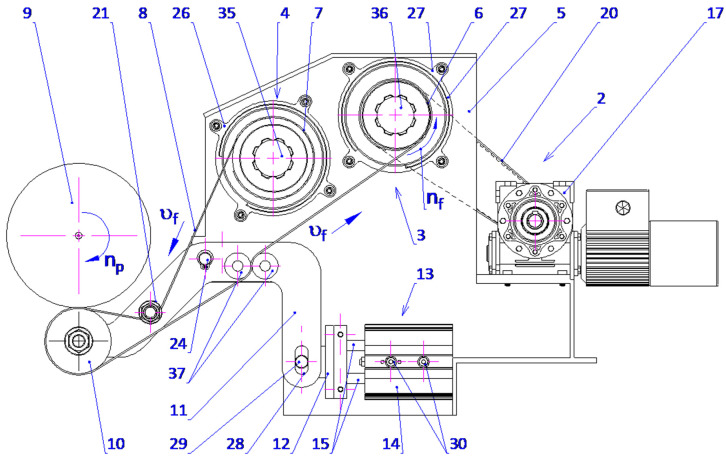
The lever and pressing mechanism from the workpiece side view.

**Figure 16:**
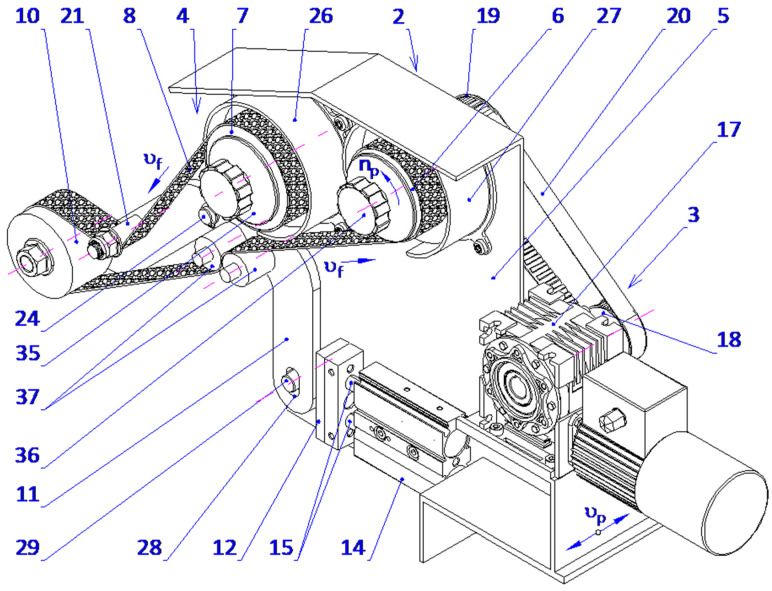
The lever and pressing mechanism from the isometric view.

The attachment assembly includes a pressing roller carried on a double-arm lever pivoted to an axis rigidly secured to the main structure of the abrasive film device. The abrasive material is cast onto the pressing roller. In the operating position, it submerges the bottom half of the workpiece into the processing liquid. Such a design provides sufficient and effective contact between the work surface and the abrasive layer in accordance with the set requirements. Compressive force of the abrasive film onto the surface of the workpiece is produced through a lever mechanism that has a horizontally aligned double-acting pneumatic actuator mounted to the lateral side of the body of the mechanism. The piston rod of the actuator is rigidly attached to a foot by means of a pin that extends through an elliptical aperture of the double-arm lever. The pressing roller is mounted at one end of the lever and is free to rotate about its axis. The force, F, applied is calculated from the piston rod elongation, developing torque on the lever and consequently a controlled force of the abrasive film on the work surface. Two compression rollers are mounted ahead of the drive roller. The compression rollers are attached on the pivots of a double-arm lever mechanism, which compresses excess processing fluid and then returns it to the reservoir. One of the rollers has a compliant surface, preferably made of rubber, whilst the other is fixed. The second roller may move radially with respect to the fixed one, both inwards and outwards. It is this mobility that makes fluid extraction more effective. The proposed solution is illustrated in the figures, where Figure 13 shows the technological setup in a side view, Figure 14 presents the technological setup in an isometric view, and Figure 15 and Figure 16 depict the abrasive film feed system in a side view and an isometric view, respectively.

The list of designations used in this study includes the following components: the microfinishing attachment (1), abrasive film feed system (2), tape drive assembly (3), abrasive film tape (4), housing of the abrasive film system (5), abrasive film drive roller (6), brake roller—film storage (7), tape braking assembly (8), workpiece—shaft (9), pressing roller (10), double-arm lever (11), foot (12), pneumatic actuator (13), cylinder (14), piston rod (15), cross slide (16), gear motor (17), driving pulley (18), driven pulley (19), toothed belt (20), guiding roller (21), tank (22), processing fluid (23), axis of the double-arm lever (24), machine bed (25), spool storage cover (26), drive roller cover (27), oval hole (28), pin (29), screw (30), processing fluid level (31), tailstock (32), machine bed (33), workpiece holder (34), lateral film storage cover (35), lateral drive roller cover (36), squeezing roller (37), machine center (38), and pressing roller axis (39). The microfinishing attachments are mounted on the cross slides of the universal lathes or on specialized machines designed for finishing with abrasive films. The head (1) is equipped with an abrasive film feed system (2) consisting of two rollers with horizontal axes, one serving as the film storage (7) and the other as the drive roller (6), which is mechanically driven to collect the used abrasive film (8). Additionally, the head features a pressing roller (10) and a guiding roller (21) mounted rotatably on the end of a double-arm pivoting lever (11), which is slidably connected to a double-acting pneumatic actuator (13).

In this actuator, the piston rod (15) is connected to a foot (12) equipped with a pin (29) that slides within an oval hole (28) in the double-arm lever (11). The pressing roller (10) is mounted at the end of the lever on an axis (39). The attachment also includes a tape drive assembly (3) and a tape braking assembly (4). The drive for the tape assembly (3) is transmitted from a gear motor (17) through a belt transmission with pulleys (18 and 19) and a toothed belt (20) to the shaft on which the abrasive film drive roller (8) is mounted. Furthermore, two squeezing rollers (37) are positioned in front of the drive roller (6) to remove excess processing fluid, which flows back into the tank (22). One of the squeezing rollers has a surface coated with an elastic material, preferably rubber. While one roller is fixed in place, the other can move radially towards or away from the fixed roller, enhancing fluid removal efficiency. The workpiece (9) is rotated at a specific speed, for example, by the lathe spindle, while the head (1) moves back and forth at a feed rate determined by the cross slide of the lathe (16). The abrasive film (8) advances at a speed governed by the drive roller (6). As the piston rod (15) of the pneumatic actuator (13) extends, it causes the double-arm lever (11) to rotate, pressing the abrasive film (8) against the surface of the shaft (9) and the elastic pressing roller (10). The combined movements of the workpiece (9), the head (1), and the abrasive film (8) result in a helical trajectory of the abrasive grains on the processed surface. A change in the feed direction reverses the helical pattern, creating intersecting machining marks, which are beneficial for surface finishing. This proposed solution is expected to optimize the utilization of abrasive films, enhance machining efficiency, and improve overall process performance by submerging the processing zone in a fluid environment. The immersion of the processing zone limits the influence of the surrounding atmosphere on the physical phenomena in the micro-cutting zone and isolates the effects of the processing fluid’s components on the formation of the workpiece surface. The technological setup also enables the finishing of cylindrical and conical surfaces of holes, which are mounted in a rotating chuck, for example, on a lathe.

The proposed method further improves the effectiveness of using abrasive film to increase machining efficiency and raise the quality of the process as a whole by immersing the processing area in a fluid medium. Thus, the immersion of the area under processing in a fluid environment decreases the influence of the ambient atmosphere on physical phenomena occurring in the micro-cutting zone. Therefore, it reduces the influence of the components in the processing fluid on workpiece surface evolution and assures a controlled and homogeneous finishing process. The use of the medium of the processing fluid, therefore, improves surface quality by creating stable conditions for microfinishing, improving heat dissipation and lubrication. All these features are strictly necessary in any case when it comes to reducing abrasive film wear, increasing the life of a tool, and improving the economic feasibility of an operation. This is a giant stride in the field of microfinishing where mechanical innovation meets improved process control. This type of finishing can be performed in a graphite suspension.

The integration of a submerged processing zone in the microfinishing process is a significant improvement in surface finishing technology with the use of abrasive films. The most significant advantages related to the process are increased process stability, improved heat dissipation, and better lubrication in the machining zone. The use of a processing fluid significantly reduces temperature and frictional forces, leading to reduced tool wear and a more uniform surface finish. The presence of the medium prevents the influence of atmospheric conditions upon the physical process in the zone of micro-cutting and helps to ensure greater control over the generation of the surface. In addition, the use of a liquid medium prevents abrasive particle loading as well as stripping of the removed material, thereby resulting in higher process efficiency and capability to produce surfaces with a lower roughness value.

The second major advantage of this technology is its capability to improve the quality of the surface in tandem with the reduced use of abrasive films. The life of the abrasive particles is increased, which leads to lower costs of operation and improved process efficiency. The integration of an immersed processing zone in cylindrical surface microfinishing greatly improves process stability and quality, lengthens tool life, and minimizes abrasive material usage. The process is a significant step forward towards accurate and cost-effective surface finishing, especially for high-quality and repetitive machining parameter requirements in applications.

### 3.4. Microfinishing with Abrasive Films as an Integrated Process with the Application of a Graphite Layer on the Machined Surface

**Figure 17:**
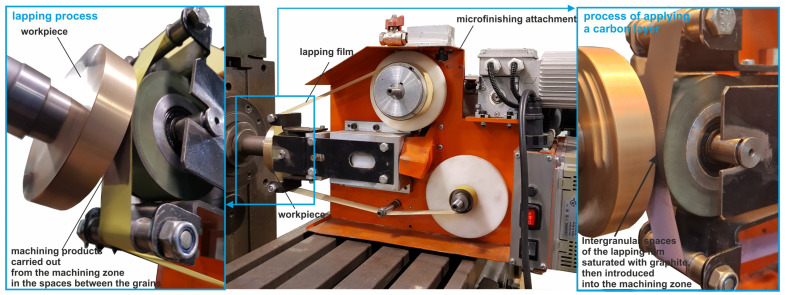
Research setup for the micro-finishing process, with the integrated simultaneous application of thin carbon layers [55].

The authors have worked out and patented a new method, which combines microfinishing with the application of carbon layers onto machined surfaces [58]. In this way, the presented method joins surface smoothing and carbon texturing in one step—a substantial step forward within the field of surface treatment technologies. The presented technique responds to the growing tendency for efficient and effective surface modification in many industries. The authors used graphite-impregnated abrasive films to smooth the soda–lime glass and tin–bronze alloy surfaces and deposit carbon films in the same process, ensuring the effectiveness and feasibility of their approach [59,60]. This technique not only reduces the roughness of the surface but also increases the durability and wear resistance of the treated surfaces. While previous studies have focused on the carbon coating and surface finishing techniques as separate processes, this is the first time that these two methods have been combined into a single process. This ongoing research in the area of surface engineering and tribology has indicated several advantages of carbon coatings, including reduced friction and wear. However, the idea to adapt carbon deposition to the microfinishing process is quite new and may revolutionize surface treatment methods in the near future. The results confirm that graphite-impregnated abrasive films can deposit an even carbon layer and, simultaneously, enhance the smoothness of the surface (Figure 17).

The bifunctional concept makes the preparatory procedures easier and improves the quality and lifetime of expensive surfaces. The very thin graphite films formed are especially advantageous in systems that may require long-term inactivity or low-maintenance operations, such as in cases where kinematic joints are not accessible, military equipment, or systems used in vacuum and low-temperature conditions. This new technology opens entirely new opportunities to efficiently realize multifunctional surface treatments for a broad field of applications.

## 4. Summary and Conclusions

The following research has found an advanced state-of-the-art microfinishing technology mainly on external cylindrical surface. This research proposes tremendous improvement in productivity and precision of machining by integrating abrasive film processing with advanced mechanical systems. The adopted mechanism, which relies on pivoting pressing assembly along with symmetrical abrasive film feed systems, allows for dual-zone processing, which enhances force distribution, thus minimizing waste. Submerging the processing zone in a liquid medium simplifies the control of parameters in microfinishing and improves surface quality, raising the tool’s lifespan.

One of the major novelties is combining microfinishing with carbon layer deposition using graphite-impregnated abrasive films; This bifunctional process assures the smoothing of the surface and accomplishing carbon texturing simultaneously, enhancing surface durability and wear resistance. In particular, such a feature brings clear benefits to systems with low-maintenance requirements or use under extreme conditions, like military applications, vacuum, or low-temperature use. This finding enables the modification of surface treatment procedures and thus paves the way to new applications of multifunctional surface engineering relevant to various industrial applications.

- The proposed dual-zone microfinishing setup dramatically improves the efficiency of machining, as it ensures a balanced distribution of forces and reduces mechanical deformation. The introduced symmetrical feed system of the abrasive film is effective for processing cylindrical surfaces with high precision and efficiency;
- The conditions of microfinishing are controlled by submerging the processing area in a fluid medium that enhances lubrication, facilitates heat dissipation, and improves surface quality. This is especially true for those procedures requiring accurately controlled finishing conditions, such as components or surfaces with very tight quality tolerances;
- Combining microfinishing with carbon layer deposition in one operation allows for simultaneous surface refinement and improvement in durability. This meets the requirement of better wear resistance and a reduction in friction, thus becoming very useful in systems running under rough conditions;
- Graphite-impregnated abrasive films easily and efficiently reduce surface roughness while depositing uniform layers of carbon. This substantially improves the durability and reliability of the treated surface, a fact that becomes particularly important with systems that have high wear resistances.

## 5. Patents

The article presents the results related to three patents owned by the authors of the publication [55,56,57,58].

## Data Availability

The original contributions presented in the study are included in the article, further inquiries can be directed to the corresponding authors.
